# Pharmacokinetics of florfenicol and its metabolite florfenicol amine in the plasma, urine, and feces of fattening male donkeys following single oral administration

**DOI:** 10.3389/fvets.2023.1314029

**Published:** 2024-01-04

**Authors:** Shijie Liu, Yanxin Guo, Honglei Qu, Yanjie Dong, Shancang Zhao, Tianze Fu, Ruifen Kang, Jie Cheng, Shimeng Huang, Lihong Zhao, Qiugang Ma

**Affiliations:** ^1^State Key Laboratory of Animal Nutrition, College of Animal Science and Technology, China Agricultural University, Beijing, China; ^2^National Engineering Research Center for Gelatin-Based Traditional Chinese Medicine, Dong-E-E-Jiao Co., Ltd., Liaocheng, China; ^3^Shandong Academy of Agricultural Sciences, Jinan, China

**Keywords:** florfenicol, florfenicol amine, pharmacokinetics, donkey, urinary excretion, antibiotic

## Abstract

Florfenicol (FF) is a commonly used antibacterial agent in animals. We investigated the pharmacokinetics of FF and its metabolite florfenicol amine (FFA) in donkeys. Donkeys were administered FF (30 mg/kg bodyweight, p.o.). Pharmacokinetic parameters were calculated using a non-compartmental model. The FF (FFA) pharmacokinetics parameters were characterized by along elimination half-life (*t*_1/2 kz_) of 5.92 h (15.95 h), plasma peak concentration (*C*_max_) of 0.13 μg/mL (0.08 μg/mL), and the time taken to reach *C*_max_ (*T*_max_) of 0.68 h (0.72 h). The area under plasma concentration-time curve and mean residence time of FF (FFA) in plasma were 1.31 μg·mL^−1^·h (0.47 μg·mL^−1^·h) and 10.37 h (18.40 h), respectively. The *t*_1/2 kz_ of FF and FFA in urine was 21.93 and 40.26 h, and the maximum excretion rate was 10.56 and 4.03 μg/h reached at 25.60 and 32.20 h, respectively. The respective values in feces were 0.02 and 0.01 μg·h^−1^ reached at 33.40 h. The amount of FF and FFA recovered in feces was 0.52 and 0.22 μg, respectively. In conclusion, FF (FFA) is rapidly absorbed and slowly eliminated after a single oral administration to donkeys. Compared to FF, FFA was more slowly eliminated. FF (FFA) is mostly excreted through urine.

## 1 Introduction

Florfenicol (FF) is a fluorinated derivative of thiamphenicol. It is a valuable synthetic reagent used to treat various antibacterial infections in animals, widely used in veterinary clinics. FF exerts its antibacterial effect by inhibiting protein synthesis at the prokaryotic ribosome. However, compared with thiamphenicol and chloramphenicol, FF is much less susceptible to bacterial deactivation as a result of fluorine substituting a hydroxyl group (1, 2). Considering its safety and high therapeutic potency, FF is used frequently for treating infections induced by *Escherichia coli*, *Salmonella typhimurium*, *Staphylococcus aureus*, *Streptococcus suis*, *Pasteurella multocida*, *Manheimia haemolytica* and *Actinobacillus pleuropneumoniae* (3, 4).

FF is mainly used for large ruminants in the treatment of bovine respiratory diseases caused by etiological agents such as *Pasteurella multocida*, *Mannheimia haemolytica*, or *Histophilus somni*. Florfenicol products are also intended for therapeutic use in acute interdigital necrobacillosis, presenting good antimicrobial activity against *Fusobacterium necrophorum* and *B. melaninogenicus* (5), but also in the case of keratoconjunctivitis produced by *Moraxella bovis* (6). Additionally, a study was identified that tested the efficacy of florfenicol in its therapeutic approach to caseous lymphadenitis in sheep and goats caused by *Corynebacterium pseudotuberculosis*. A comparison of the control groups and those who received florfenicol therapy showed an improvement in clinical scores, suggesting the effective treatment and maintenance of remission in caseous abscesses (7). In pigs, FF is used for the treatment of bacterial respiratory diseases caused by agents such as *Actinobacillus pleuropneumoniae*, *Pasteurella multocida*, *Bordetella bronchiseptica*, and *Salmonella choleraesuis* (6, 8). A recent study presented florfenicol to be a reliable option for the treatment of arthritis in pigs caused by *Streptococcus suis* (9). The pharmacokinetics of FF has been extensively studied in cattle (10, 11), pigs (12), rabbits (13), dogs (14), llamas (15), sheep (16), chickens (17–19), turkeys (20), ducks (21), and fish (22), but pharmacokinetic studies in donkeys have not been reported.

As known, donkeys are widely used in various aspects of social life, as important tools for transportation and private riding. As livestock, they are also widely used in the food processing industry. Donkey products, such as donkey meat and skin, play an important role in the catering industry and healthcare industry. Donkeys are also frequently infected with diseases, but there are few reports on effective drugs for treating donkey diseases. Based on the effectiveness of FF in other animals, it may be possible to use it as an effective drug for treating donkey diseases, and therefore pharmacokinetics needs to be carried out to further understand the metabolism of FF in donkeys, and provide a theoretical basis for the use of FF in production practice.

However, few studies have been carried out on the pharmacokinetics of FF in equine species. Designing species-specific dosing protocols and identifying the absorption, distribution, metabolism or elimination of FF in such studies is very important. Herein, we studied the pharmacokinetics of FF in the plasma, urine, and feces of donkeys.

## 2 Materials and methods

### 2.1 Chemicals and reagents

The concentration of florfenicol standard in methanol is over 99.0% which was purchased from the Research and Monitoring Institute of Environmental Protection (Ministry of Agriculture, China). And florfenicol used in this study was provided by Zhongmu Nanjing Animal Pharmaceutical Co., Ltd. (Nanjing, China).

### 2.2 Animals and treatment

The study was allowed by the Laboratory Animal Welfare and Animal Experimental Ethical Committee of China Agricultural University (No. AW80803202-1-4). Five 13 months-old male donkeys were selected and housed individually in metabolic cages. The donkeys were allowed to adapt to their surroundings for 7 days and fasting overnight before the experiment. Then, the body weight (BW) of donkeys was measured (173.20 ± 14.39 kg). The samples of blank blood, urine and feces were collected at 6 h prior to the start of the experiment. At the start of the experiment, the FF solution was orally administered through an esophageal tube in a single dose of 30 mg/kg BW. The animals had free access to water during the experimental period. Each donkey received their individual ration of concentrates twice daily (7 am and 5 pm), with weighing 1.5 kg/d per donkey. The composition and nutrient levels of concentrate was reported in Table 1. Meanwhile, each donkey was fed the Leymus chinensis hay four times a day (8 am, 12 am, 4 pm and 8 pm), with averagely 2.1 kg/d per donkey. The analyzed values of dry matter (DM), crude protein (CP), crude fiber (CF), neutral detergent fiber (NDF), acid detergent fiber (ADF), ether extract (EE) and ash were 90.64, 7.78, 35.32, 68.17, 44.19, 1.10 and 5.15%, respectively in the hay.

**Table 1:** Composition and nutrient levels of concentrate.

Ingredients	Composition (%)	Nutrients	Levels^b^
Corn	45.00	Dry matter (DM)	87.56
Soybean meal	15.00	DE/MJ·kg^−1^	10.79
Wheat bran	20.00	Crude protein (CP)	22.18
Corn germ meal	10.00	Crude fiber(CF)	6.26
Peanut meal	5.00	Ether extract (EE)	1.38
Premix^a^	5.00	Lysine	1.12
Total	100.00	Calcium	1.07
		Phosphorus	0.65

a The premix provided the following per kg of concentrate diets VA 12,000 IU, VD3 1,500 IU, VE 40 mg, VK3 3 mg, VB1 2 mg, VB2 4 mg, VB6 4 mg, VB12 0.01 mg, pantothenic acid 20 mg, nicotinic acid 30 mg, folic acid 1.7 mg, biotin 0.3 mg, Cu 20 mg, Zn 80 mg, Fe 100 mg, Mn 80 mg, I 0.6 mg, Se 0.2 mg.

b DE was a calculated value, while the others were measured values; except for DM, the others were air-dry basis.

### 2.3 Blood, plasma, feces and urine collection

Blood was collected from the jugular vein in heparin anticoagulant tubes before administration (0 h) and 0.08, 0.25, 0.42, 0.58, 0.75, 1.0, 1.5, 2.0, 2.5, 3, 4, 5, 6, 8, 10, 12, 24, 36, 48, 72, 96, and 144 h after administration. The blood samples were centrifuged at 3,000 rpm for 20 min at 4°Cto obtain plasma and were stored at −20°C until analyses. The urine and feces were collected and weighed every 6 h. After each collection, the weight of feces and the volume of urine were recorded and stored into 50 mL centrifuge tubes for subsequent analysis.

### 2.4 Determination of FF and FFA

The concentrations of FF and its metabolite FFA were determined using high performance liquid chromatography (HPLC) tandem mass spectrometry detection. The assay was referenced to previous reports (9).

Briefly, 0.1 mL of FF and FFA standard stock solution, respectively, were placed in a 10 mL volumetric flask. The volume was made up to 10 mL with methanol. A mixed standard working solution of concentration 1 μg/mL was prepared. Then, 0.1 mL of FF-d3 and FFA-d3 isotope internal standard stock solution, respectively, were placed in a 10 mL volumetric flask. The volume was made up to 10 mL with methanol. A mixed internal standard working solution of FF-d3 and FFA-d3 was prepared. An appropriate amount of the standard working solution of FF and FFA was measured accurately. The control solution was diluted with acetonitrile to make concentrations of 5, 10, 20, 50, 100, and 200 ng/mL. The internal standard solution was mixed with a concentration of 50 ng/mL. A series of control solutions from low concentrations to high concentrations was created. A standard curve was created according to the ratio of the obtained peak area to the concentration of the corresponding control solution. We calculated the regression equation and correlation coefficient. The sample solution and control solution of 50 ng/mL (based on the FF concentration) were used for calibration at a single point. The response values of FF and FFA in the control solution and sample solution had to be within the linear range of detection for the instrument. During determination of the sample solution, the reference solution was injected after 10 single batches of samples to facilitate accurate quantification. Subsequently, we weighed 1 g of blood, urine, or feces, respectively, and placed them in 50 mL centrifuge tubes. Then, we added 50 μL of mixed internal standard working solution. Next, we added 5.0 mL of water and 0.5 mL of ammonia solution, and vortex-mixed for 1 min. Then, we added 10.0 mL of acetonitrile and vortex-mixed immediately for 1 min. The next step was ultrasonic agitation for 20 min, followed by addition of NaCl (3 g) and stirring for 1 min. Next, we undertook ultrasonic agitation for 10 min, followed by centrifugation at 8,000 × *g* for 5 min at room temperature. And then, 1 mL of the supernatant was transferred to a test tube and the extraction procedure was repeated, and make the liquid flow out at a constant speed drop by drop through HLB columns (Tianjin Alta Technology, Tianjin, China), lastly put it on the HPLC-MS/MS for determination. The mobile phase was 5% acetonitrile (phase A) and 95% formic acid water (phase B). The flow rate was maintained at 1.0 mL/min.

### 2.5 HPLC method validation

The method was validation for linearity, sensitivity and recovery in plasma, urine and feces. Standard curves were constructed from the concentrations of 5, 10, 20, 50, 100, and 200 ng/mL FF or FFA in plasma, urine, and feces, respectively, with their peak areas. The limit of detection (LOD) and limit of quantitation (LOQ) were calculated. Recoveries were calculated by the ratio of the peak areas of different concentrations of FF or FFA obtained from spiked samples of plasma, urine, and feces to the peak area of the corresponding FF or FFA standard working solution.

### 2.6 Pharmacokinetics analyses

We undertook a non-compartmental approach based on the combined linear trapezoidal rule using WinNonlin 8.3.5.0 (Pharsight, Mountain View, CA, United States). The *λz* was a first-order rate constant associated with the terminal (log linear) segment of the curve. It was estimated by linear regression of terminal data points. The terminal elimination half-life (*t*_1/2 kz_) was calculated as 0.693/*λz*. The area under the plasma concentration-time curve (AUC) was calculated using the trapezoid method. The area under the rate curve (AURC) was calculated as the product of time and excretion rate.

The peak plasma concentration (*C*_max_) of the drug and the time to reach the peak plasma concentration (*T*_max_) were determined from individual plasma concentration-time curves. The ratio of AUCs of FFA and FF was calculated. The concentrations of FF and FFA in plasma, urine, and feces versus time for each donkey were reported as the mean ± SD at each time point.

## 3 Results

LC/MS for simultaneous determination of FF and FFA was rapid with a high degree of reproducibility. We obtained calibration curves of FF (FFA) for plasma, urine and fecal matrix with *R*^2^ of 0.9988 (0.9992), 0.9971 (0.9980) and 0.9962 (0.9977), respectively. The mean inter-day precision and intra-day precision was <20%. Accuracy ranged from 80.9 to 113.5%. The limit of detection (LOD) and limit of quantitation (LOQ) were 0.5 μg/kg and 1.0 μg/g or μg/mL for FF and FFA that is calculated according to a signal-to-noise ratio >3 and >10. These validation parameters indicate that this assay method used in this study is accurate and precise.

**Figure 1:**
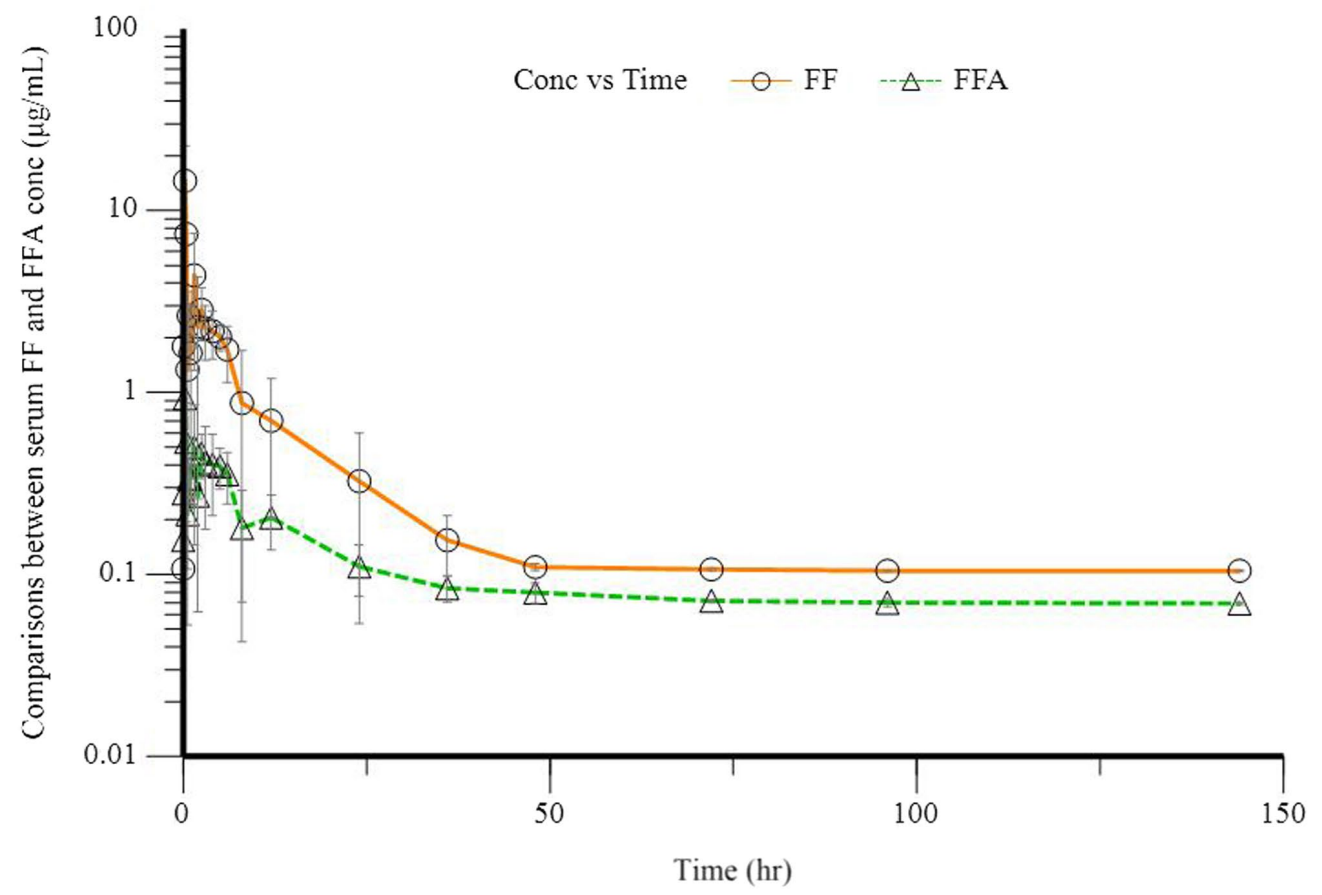
Plasma concentration of florfenicol (FF, O) and florfenicol amine (FFA, Δ) in five donkeys after a single oral dose of 30 mg/kg bodyweight.

The mean serum concentration of FF and FFA in these five donkeys is shown in Figure 1. The pharmacokinetics parameters for FF after administration (30 mg·kg^−1^ BW, p.o.) to donkeys are shown in Table 2. After administration of FF, the *C*_max_ was 0.13 ± 0.02 μg·mL^−1^, *T*_max_ was 0.68 ± 0.09 h, and elimination half-life was 5.92 ± 3.25 h. The AUC of FF was 1.31 ± 0.46 μg·mL^−1^·h, and the mean residence time for FF was 10.37 ± 4.80 h. FFA was detected in all donkeys after administration. The *C*_max_ of FFA was 0.08 ± 0.01 μg·mL^−1^, *T*_max_ was 0.72 ± 0.72 h, and elimination half-life was 15.95 ± 14.04 h. The AUC of FFA was 0.47 ± 0.11 μg·mL^−1^·h, and the mean residence time for FFA was 18.40 ± 13.02 h. Plasma results can show that FF and FFA are absorbed quickly and eliminated slowly in donkeys.

**Table 2:** Pharmacokinetic parameters of florfenicol in the plasma of five donkeys after single oral administration of 30 mg/kg bodyweight.

Parameter (unit)	Donkey	BW (kg)	*λz* (1·h^−1^)	*t*_1/2 kz_ (h)	*T*_max_ (h)	*C*_max_ (μg/mL)	AUC_0–∝_ (μg·mL^−1^·h)	MRT (h)
Florfenicol	Boris	156	0.23	3.00	0.75	0.14	0.92	6.22
	John	172	0.22	3.12	0.75	0.14	0.98	7.11
	Jack	196	0.09	7.99	0.75	0.14	1.72	12.66
	Robin	170	0.14	5.00	0.58	0.10	1.01	8.15
	Donald	172	0.07	10.48	0.58	0.11	1.91	17.72
	Mean (SD)	173.20 ± 14.39	0.15 (0.08)	5.92 (3.25)	0.68 (0.09)	0.13 (0.02)	1.31 (0.46)	10.37 (4.80)
Florfenicol amine	Boris	156	0.06	11.07	1.50	0.08	0.62	16.96
	John	172	0.06	12.22	0.25	0.08	0.41	12.44
	Jack	196	0.08	8.64	1.50	0.06	0.53	13.07
	Robin	170	0.10	7.03	0.25	0.07	0.34	8.46
	Donald	172	0.02	40.80	0.08	0.09	0.48	41.06
	Mean (SD)	173.20 ± 14.39	0.06 (0.03)	15.95 (14.04)	0.72 (0.72)	0.08 (0.01)	0.47 (0.11)	18.40 (13.02)

Values are the mean ± SD. BW, bodyweight; *λz*, first order rate constant associated with the terminal portion of the curve; *t*_1/2*λz*_, terminal half-life; AUC, area under the curve; *T*_max_, time needed to reach the maximum concentration in plasma; *C*_max_, maximum concentration in plasma; MRT, mean residence time.

**Figure 2:**
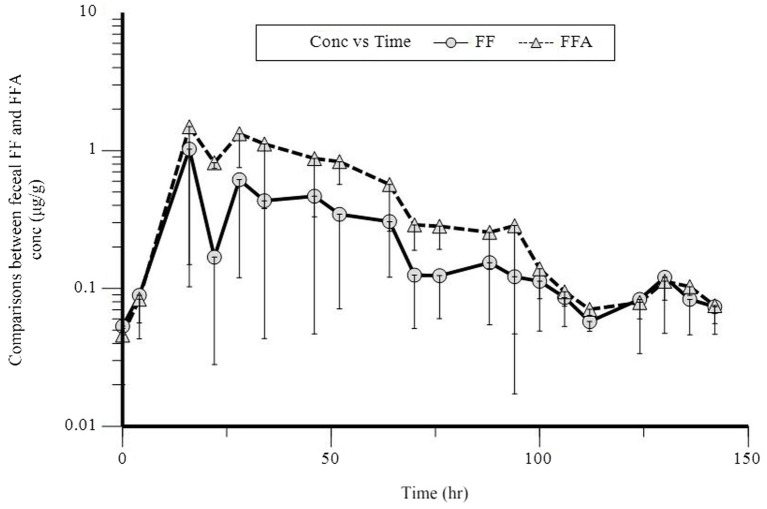
Semilogarithmic plot of florfenicol (FF, O) and florfenicol amine (FFA, Δ) urinary concentration vs. time following a single oral dose at 30 mg/kg bodyweight in donkeys.

In a semi-logarithmic plot, the relationship between the urinary concentration of FF and FFA and time was evaluated (Figure 2). Table 3 illustrates the individual and mean pharmacokinetics parameters of FF and FFA in urine. FF was excreted mainly unchanged. The urinary concentration of FF and its metabolite FFA was 123.08 ± 51.71 and 63.62 ± 28.76 μg·L^−1^, respectively. The *t*_1/2 kz_ of FF and FFA in urine was 21.93 ± 3.42 and 40.26 ± 26.00 h, respectively. The maximum excretion rate of FF and FFA was 10.56 ± 6.41 and 4.03 ± 0.59 μg·h^−1^, and was reached at 25.60 ± 11.70 and 32.20 ± 9.86 h, respectively.

**Table 3:** Amount of florfenicol and florfenicol amine recovered in urine after single oral administration to donkeys at 30 mg/kg bodyweight.

Parameter (unit)	Donkey	*λz* (1·h^−1^)	*t*_1/2 kz_ (h)	*T*_max rate_ (h)	Max rate (μg·h^−1^)	Amount recovered (μg)	AURC (μg)
Florfenicol	Boris	0.02	34.59	25.00	7.82	116.66	358.70
	John	0.02	31.71	25.00	12.03	311.62	454.08
	Jack	0.36	1.94	43.00	6.29	87.50	149.54
	Robin	0.02	28.86	49.00	3.04	87.80	147.82
	Donald	0.01	54.16	25.00	5.70	159.94	448.44
	Mean (SD)	0.03 (0.01)	21.93 (3.42)	25.60 (11.70)	10.56 (6.41)	123.08 (51.71)	153.93 (43.59)
Florfenicol amine	Boris	0.02	44.84	37.00	3.05	34.43	96.14
	John	0.02	32.03	19.00	4.02	110.75	151.46
	Jack	0.01	79.44	43.00	4.60	54.61	173.67
	Robin	0.02	37.47	37.00	4.22	51.57	76.16
	Donald	0.09	7.52	25.00	4.28	66.74	142.60
	Mean (SD)	0.03 (0.03)	40.26 (26.00)	32.2 (9.86)	4.03 (0.59)	63.62 (28.76)	128.01 (40.47)

Values are the mean ± SD. *λz*, first order rate constant associated with the terminal portion of the curve; *t*_1/2 kz_, terminal half-life; AURC, area under the rate curve; *T*_max rate_, time needed to reach the maximum rate; Max rate, maximum excretion rate; amount-recovered, amount of drug excreted in urine.

**Figure 3:**
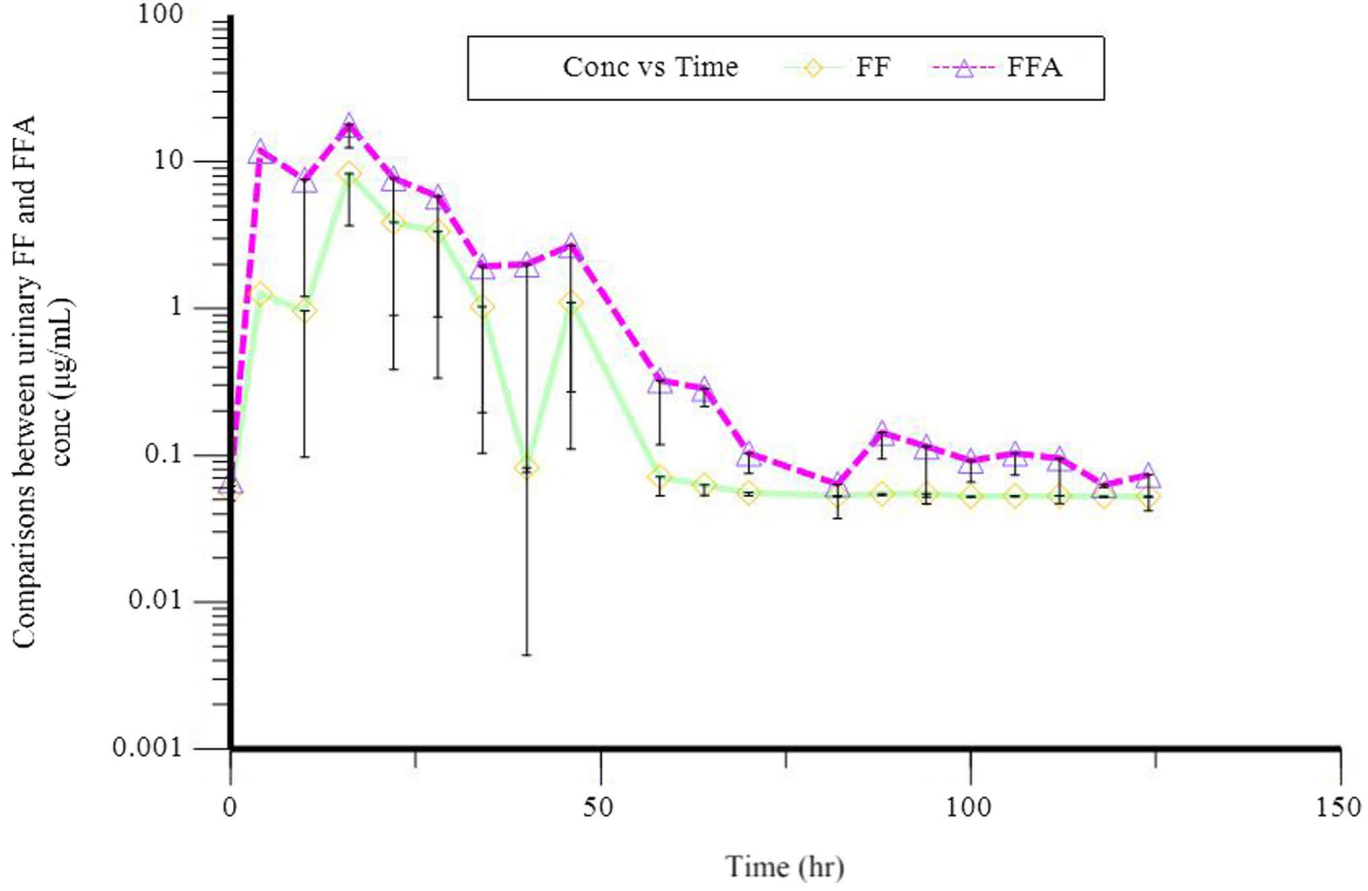
Semilogarithmic plot of florfenicol (FF, O) and florfenicol amine (FFA, Δ) fecal concentration vs. time after a single oral dose of 30 mg/kg bodyweight in donkeys.

In a semi-logarithmic plot, the relationship between the fecal concentration of FF and FFA and time was evaluated (Figure 3). Table 4 presents the individual and mean pharmacokinetics parameters of FF and FFA in feces. The maximum excretion rate of FF and FFA in feces was 0.02 ± 0.01 and 0.01 μg·h^−1^, respectively, and was reached in 33.40 h. The recovery of FF and FFA in feces was 0.52 ± 0.17 and 0.22 ± 0.05 μg, respectively. The area under the feces concentration versus time curve (AURC) of FF and FFA was 0.81 ± 0.36 and 0.34 ± 0.18 μg, respectively. The amount of FF (FFA) excreted through feces is less compared to urine, and it is seen that FF and FFA are mainly excreted through urine.

**Table 4:** Pharmacokinetic parameters calculated from the feces of donkeys administered florfenicol (30 mg/kg bodyweight, p.o.).

Parameter (unit)	Donkey	*λz* (1·h^−1^)	*t*_1/2 kz_ (h)	*T*_max rate_ (h)	Max rate (μg·h^−1^)	Amount-recovered (μg)	AURC (μg)
Florfenicol	Boris	0.017	41.91	25.00	0.012	0.605	0.831
	John	0.009	77.76	43.00	0.012	0.606	1.363
	Jack	0.011	64.83	43.00	0.011	0.222	0.400
	Robin	0.024	29.17	31.00	0.037	0.521	0.624
	Donald	0.011	65.67	25.00	0.012	0.647	0.837
	Mean (SD)	0.01 (0.01)	55.87 (19.77)	33.40 (9.10)	0.02 (0.01)	0.52 (0.17)	0.81 (0.36)
Florfenicol amine	Boris	0.083	8.31	49.00	0.010	0.251	0.248
	John	0.012	59.00	19.00	0.008	0.227	0.293
	Jack	0.004	168.32	43.00	0.008	0.136	0.671
	Robin	0.022	30.83	31.00	0.019	0.249	0.263
	Donald	0.051	13.52	25.00	0.009	0.228	0.242
	Mean (SD)	0.03 (0.03)	56.00 (65.84)	33.40 (12.44)	0.01 (0.00)	0.22 (0.05)	0.34 (0.18)

Values are the mean ± SD. *λz*, first order rate constant associated with the terminal portion of the curve; *t*_1/2 kz_, terminal half-life; AURC, area under the rate curve; *T*_max rate_, time needed to reach the maximum rate; Max rate, maximum excretion rate; amount-recovered, amount of drug recovered from feces.

## 4 Discussion

Few studies have been carried out on the pharmacokinetics of FF in equine species (23). In the present work, a non-compartmental model best described the time-course of FF in the plasma of donkeys following oral administration. FF was absorbed rapidly through the gastrointestinal tract with a *C*_max_ of 0.13 μg/mL at 0.68 h. The *C*_max_ of the present study was much lower than that reported in different animal species. Upon oral administration of FF (22 mg/kg BW), horses had a *C*_max_ of 13.80 mg/mL and *T*_max_ of 1.13 h (24). Pigs were orally administered FF at 20 mg/kg BW and 30 mg/kg BW, the *C*_max_ and *T*_max_ was 9.9 μg/mL and 1.50 h, 10.84 μg/mL and 1.35 h, respectively (12, 25). The *C*_max_ and *T*_max_ of rabbits was 7.96 μg/mL and 0.90 h, 15.14 μg/mL and 0.50 h, respectively, when oral administration of 20 mg/kg body weight and 30 mg/kg body weight FF (13, 26). Also, after administration of FF, the *C*_max_ (μg/mL) and *T*_max_ (h) have been reported to be 6.18 and 0.94 in dogs (14), 2.41 and 1.16 for chickens (27), 30.47 and 0.50 in geese (28). This difference may have resulted from the formulation type for different species (23) and variation in the FF dose administered (15 versus 20 versus 22 versus 30 mg/kg BW) (29).

*λz* and *t*_1/2 kz_ are constants that reflect the rate of drug elimination from the body, and there is a reciprocal relationship between them. Oral administration of FF (20 mg/kg BW) led to a *t*_1/2 kz_ (h) of 10.0 in pigs (12), 1.42 in rabbits (13), 1.24 in dogs (14), 2.43 in geese (28), 21.93 in one group of chickens (17), 9.0 in another group of chickens (27). Oral administration of FF (30 mg/kg BW) led to a *t*_1/2 kz_ (h) of 12.39 in pigs (12), 2.57 in rabbits (26), 1.67 in chickens (18), 3.76 in broiler turkeys (20), and 2.77 in ducks (21). In our study, the *t*_1/2 kz_ for FF in the plasma of donkeys was 5.92 h, which differed to the values for the animals mentioned above. Inter-species variability affects pharmacokinetics parameters, and it is a major issue in veterinary pharmacology (23). Age, sex, breed, health status, or gene polymorphisms have also been shown to influence pharmacokinetics indices in different pharmacokinetic studies (30).

The AUC is an important index to evaluate the degree of drug absorption and drug exposure. Upon oral administration of FF at 20 mg·kg^−1^ BW, the AUC has been reported to be 53.45 mg·h·L^−1^ in Equidae (25), 132.1 μg·h·mL^−1^ in pigs (12), 23.78 μg·h/mL in rabbits (13), 22.36 mg·h/L in dogs (14), and 37.85 mg·h/L in chickens (27). Upon oral administration of FF at 30 mg/kg BW, the AUC was 65.89 μg/mL/h in pigs (12), 49.02 μg/mL/h in rabbits (13), 27.59 mg·h/L in one type of chicken, 4.15 mg·h/L in another type of chicken (18), 77.62 μg·h/mL in broiler turkeys (20), and 84.00 μg·h/mL in ducks (21). In the present work, when five donkeys were administered FF at 30 mg/kg BW, the AUC in plasma was 1.31 μg·mL^−1^·h, which indicated that the degree of drug exposure in the body was very low and very little FF was absorbed. A possible explanation may be associated with the differences in BW and animal species-related cytochrome P450 enzymes. One study revealed that an increase in BW had a significant influence on the variability of pharmacokinetics parameters within the same species (31). These results of plasma pharmacokinetic parameters suggest that donkeys appear to have low bioavailability of FF (FFA) under oral administration, but this requires subsequent in-depth studies.

In the current study, the amount of FF excreted amount in urine was 123.08 μg and the excreted amount of FFA was 63.62 μg. Similarly, the amount of FF excreted in feces was 0.52 μg and that of FFA was 0.22 μg. These data indicated that FF was excreted mainly in urine. One study found that most of a drug dose was excreted in urine as the parent form, which suggested that major clearance of the drug was in the kidneys (10), which was also evidenced in our work. When FF was administered to calves, FF was excreted in urine as the parent form (10). FF and its metabolite FFA are eliminated primarily by the kidneys through glomerular filtration. Therefore, some portion of the administered drug may be affected by first-pass metabolism or may be excreted by the kidneys without reaching the general systemic circulation (23, 29). However, FF is metabolized by cytochrome P450 3A in the liver to FFA, and the same process is assumed to occur in the kidneys (27, 32). Therefore, when using FF (FFA), in addition to considering the dosage, measures should also be considered to counteract the possible environmental pollution caused by antibiotic urinary excretion. The results of this study provide a reference for the rational use of FF in donkey clinical practice, and selecting the appropriate dosage based on pharmacokinetics will save economic costs.

## 5 Conclusion

The present study suggests that after a single oral administration of FF (30 mg/kg BW), it was rapidly absorbed and slowly eliminated in male donkeys. Most FF (FFA) was excreted through feces and urine, and the amount excreted in urine was larger than in feces.

## Data availability statement

The original contributions presented in the study are included in the article/supplementary material, further inquiries can be directed to the corresponding author.

## Ethics statement

The animal studies were approved and conducted according to the guidelines for experimental animals of the Ministry of Science and Technology (Beijing, China) and approved by the Laboratory Animal Welfare and Animal Experimental Ethical Committee of China Agricultural University (No. AW80803202-1-4, 8 August 2023). The studies were conducted in accordance with the local legislation and institutional requirements. Written informed consent was obtained from the owners for the participation of their animals in this study.

## Author contributions

SL: Investigation, Methodology, Writing – original draft. YG: Investigation, Software, Writing – original draft. HQ: Formal analysis, Investigation, Resources, Writing – review & editing. YD: Methodology, Writing – review & editing. SZ: Methodology, Writing – review & editing. TF: Investigation, Writing – review & editing. RK: Formal analysis, Methodology, Writing – review & editing. JC: Resources, Writing – review & editing. SH: Supervision, Writing – review & editing. LZ: Visualization, Writing – review & editing. QM: Conceptualization, Funding acquisition, Project administration, Supervision, Writing – review & editing.
